# Sequential multimodal management for recurrent pulmonary NUT carcinoma: a case report

**DOI:** 10.3389/fonc.2026.1834836

**Published:** 2026-05-12

**Authors:** Jincheng Su, Zirong Mi, Haizhou Yue, Dongliang Bian, Peng Zhang

**Affiliations:** 1 School of Medicine, Shihezi University, Shihezi, China; 2 Department of Thoracic Surgery, Shanghai Pulmonary Hospital, School of Medicine, Tongji University, Shanghai, China

**Keywords:** case report, immunotherapy, MTAP loss, multimodal therapy, pulmonary NUT carcinoma

## Abstract

**Background:**

Pulmonary NUT carcinoma is an ultra-rare and highly aggressive malignancy associated with extremely poor survival, especially in thoracic primary tumors. Clinically meaningful prolonged survival after postoperative locoregional recurrence without distant metastasis, particularly in a child or adolescent with primary pulmonary NUT carcinoma, has rarely been described. Here, we report a notable case managed with sequential multimodal treatment across multiple lines, resulting in 26 months of post-recurrence survival.

**Case description:**

We report a 13-year-old male initially diagnosed with stage IB primary pulmonary NUT carcinoma and treated with complete surgical resection. Twenty-two months later, he developed intrathoracic recurrence without distant metastasis. He subsequently received sequential multimodal treatment, including locoregional therapy, chemotherapy plus immunotherapy, and later anti-angiogenic therapy combined with immunotherapy and salvage chemotherapy, with transient disease control achieved at multiple time points. Molecular and immunohistochemical analyses of the primary tumor confirmed NUT positivity, PD-L1 negativity, and MTAP loss. The patient ultimately died from multiple organ failure. The post-recurrence survival, defined as the interval from confirmed postoperative recurrence to death, was 26 months, exceeding historically reported survival for thoracic NUT carcinoma.

**Conclusion:**

This case suggests that prolonged survival in recurrent pulmonary NUT carcinoma may be achievable through individualized multimodal management. Early complete resection, aggressive locoregional control, and the integration of systemic therapies (including immunotherapy-based combinations) may contribute to extended disease control. In our patient, immune checkpoint inhibitors combined with chemotherapy and multi-target inhibitor therapy provided periods of disease stabilization, highlighting the potential role of immunotherapy as part of a multimodal treatment strategy. Comprehensive molecular profiling, including assessment of MTAP status, may further inform future therapeutic options in this rare malignancy.

## 1 Introduction

NUT carcinoma (NC) is a rare and highly aggressive epithelial malignancy defined by chromosomal rearrangements involving the NUTM1 (nuclear protein in testis midline carcinoma family member 1) gene (1, 2). Although initially described as a midline carcinoma affecting adolescents and young adults, it is now recognized across a broader age spectrum and in diverse anatomical sites, including the lung (3). Primary pulmonary NUT carcinoma represents one of the most clinically challenging manifestations of this disease due to its rapid progression, early metastasis, and resistance to conventional therapies. Pulmonary NUT carcinoma is associated with an extremely poor prognosis despite multimodal treatment approaches (3, 4). Reported median overall survival (OS) for patients with NUT carcinoma across all primary sites ranges from approximately 6 to 9 months, with thoracic (including primary lung) tumors demonstrating particularly unfavorable outcomes (3, 5). Here, we report a notable case of primary pulmonary NUT carcinoma in an adolescent, initially treated by complete surgical resection, followed by postoperative locoregional recurrence without distant metastasis and managed with sequential multimodal therapy across multiple lines, achieving 26 months of post-recurrence survival. This case may provide a useful clinical reference for the management of recurrent primary pulmonary NUT carcinoma in children and adolescents. 

## 2 Case presentation

**Figure 1:**
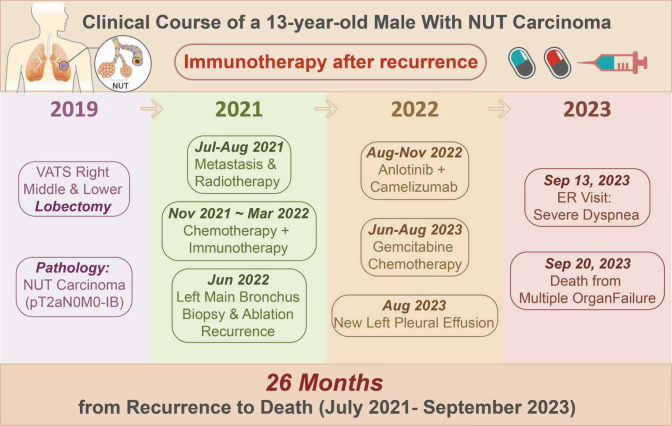
Treatment timeline of the patient.

A 13-year-old male with no smoking history or relevant family history presented with a 1-month history of cough with yellow sputum and intermittent fever (peak 39. 5 °C). Initial chest computed tomography (CT) performed at a local hospital revealed right lower-lobe atelectasis with a small pleural effusion. Bronchoscopy demonstrated an endobronchial mass in the right bronchus intermedius, and biopsy confirmed NUT carcinoma. The patient was referred to Shanghai Pulmonary Hospital for further management. Treatment timeline of the patient is as follows (**Figure 1**).

**Figure 2:**
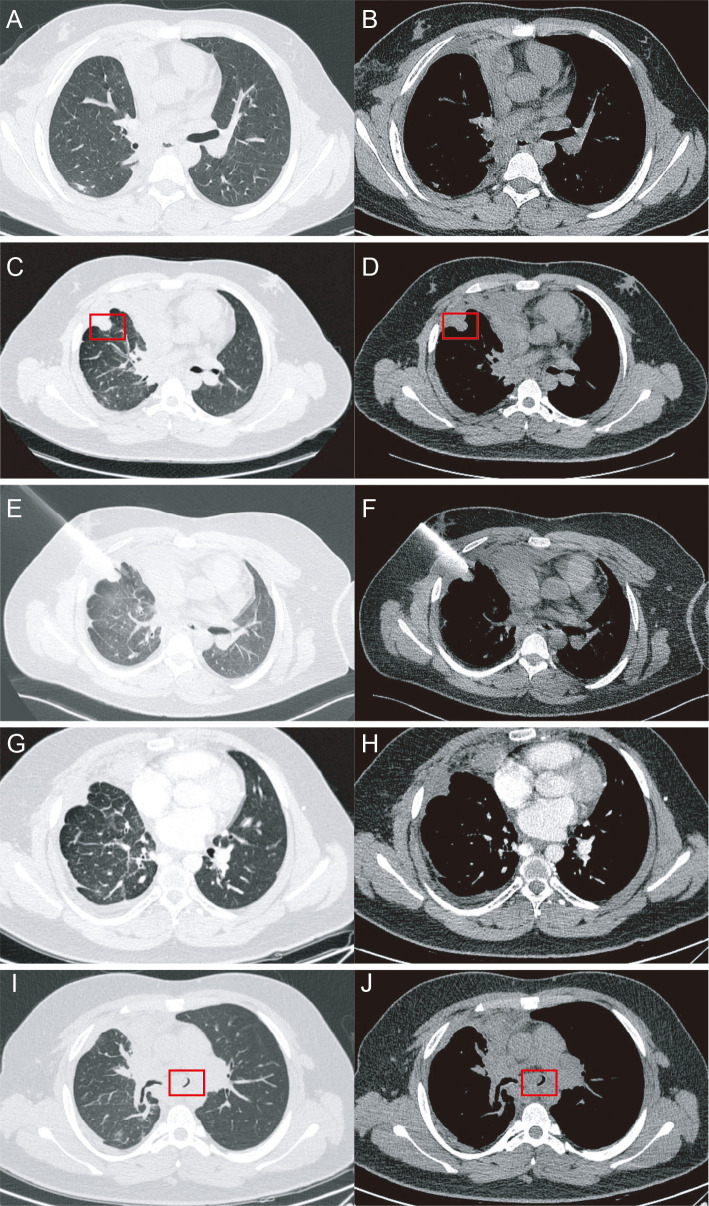
Chest CT images. 1-month re-examination after surgery **(A)**, Lung window. **(B)**, Mediastinal window). 22-month re-examination after surgery **(C)**, Lung window. **(D)**, Mediastinal window). During ablation **(E)**, Lung window. **(F)**, Mediastinal window). 3-month re-examination after ablation and radiotherapy **(G)**, Lung window. (**H)**, Mediastinal window). 3-month re-examination after nab-paclitaxel plus camrelizumab therapy [**(I)**, Lung window.**(J)**, Mediastinal window].

After multidisciplinary evaluation and exclusion of surgical contraindications, the patient underwent video-assisted thoracoscopic surgery (VATS) right middle and lower lobe sleeve resection on September 1, 2019. Pathology confirmed pulmonary NUT carcinoma with negative peribronchial lymph nodes and a final stage of pT2aN0M0 (stage IB). Immunohistochemistry showed TTF-1 (−), Napsin A (−), CK5/6 (+), p40 (+), p63 (+), NUT (+), Ki-67 (~40%), PD-L1 (E1L3N) (−), BAP1 (+), and MTAP (−). The postoperative course was uneventful and the patient was followed regularly (**Figures 2A, B**).

In July 2021, surveillance CT revealed increased right pleural nodules and suspected mediastinal lymphadenopathy (**Figures 2C, D**), consistent with locoregional recurrence. Systemic staging (brain MRI, bone ECT, and abdominal ultrasound) showed no distant metastasis. The patient underwent radiofrequency ablation (6) (RFA) of pulmonary lesions in August 2021 (**Figures 2E, F**), followed by thoracic radiotherapy with dose escalation to a total of 54Gy in 30 fractions. The best documented response to this locoregional treatment phase was transient radiographic tumor shrinkage (**Figures 2G, H**).

**Figure 3:**
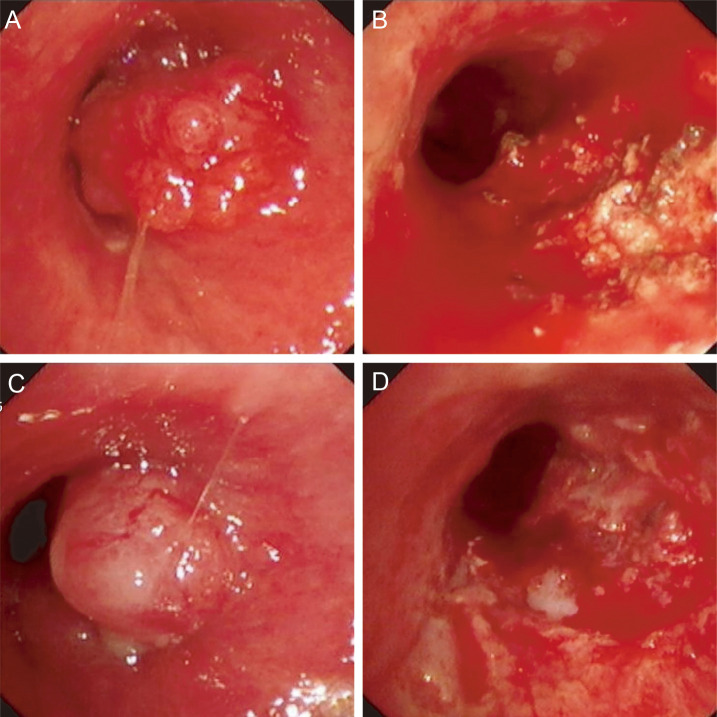
Images during bronchoscopic electrocautery ablation. First-time **(A)**, before. **(B)**, after). Second-time **(C)**, before. **(D)**, after).

From November 2021 to March 2022, the patient received five cycles of nab-paclitaxel plus camrelizumab. Nab-paclitaxel was administered at 290 mg on day 1, and camrelizumab was administered at 200 mg on day 1, every 3 weeks per cycle. The best documented response during this phase was stable disease (SD). However, on June 1, 2022, initial chest computed tomography (CT) performed at a local hospital revealed right lower-lobe atelectasis with a small pleural effusion (**Figures 2I, J**). Bronchoscopic biopsy confirmed recurrent NUT carcinoma, supported by immunophenotype (TTF-1 and Napsin A negative; p40, CK, CK5/6 positive; NUT partially positive) and FISH confirmation of NUT rearrangement. Despite two cycles of single-agent nab-paclitaxel, the endobronchial lesion progressed, consistent with progressive disease (PD), requiring repeat bronchoscopic electrocautery ablation. After the procedure, the patient experienced immediate relief of airway obstruction-related symptoms, with improvement in cough and dyspnea, indicating meaningful short-term clinical benefit in addition to local airway control (**Figure 3**).

**Figure 4:**
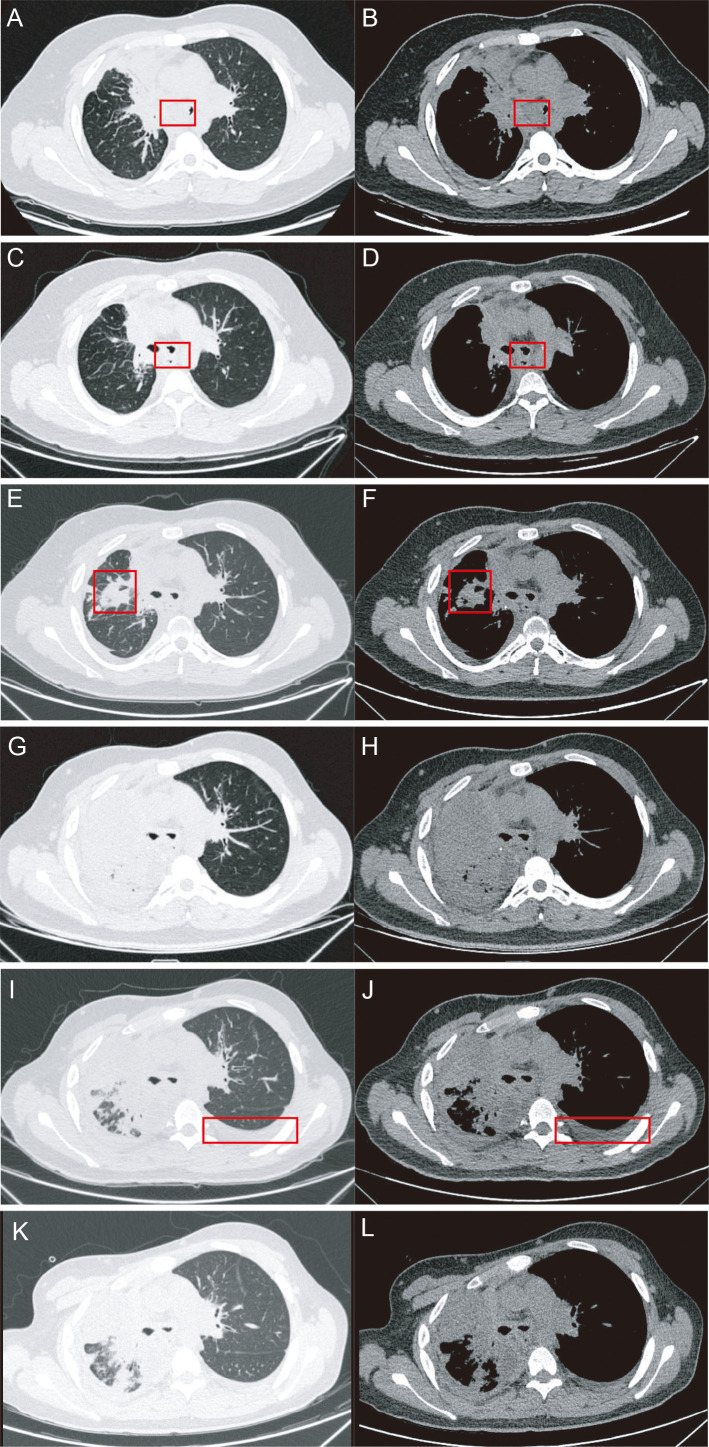
Chest CT images. Before anlotinib plus camrelizumab therapy **(A)**, Lung window. **(B)**, Mediastinal window). 3-cycle reevaluation during anlotinib plus camrelizumab therapy **(C)**, Lung window. **(D)**, Mediastinal window). 5-cycle reevaluation during anlotinib plus camrelizumab therapy **(E)**, Lung window. **(F)**, Mediastinal window). Before gemcitabine therapy **(G)**, Lung window. **(H)**, Mediastinal window). 4-cycle reevaluation during nab-paclitaxel plus camrelizumab therapy **(I)**, Lung window. **(J)**, Mediastinal window). 5-cycle reevaluation during nab-paclitaxel plus camrelizumab therapy **(K)**, Lung window. **(L)**, Mediastinal window).

Given further progression after multiple lines of therapy, oral anlotinib was initiated in August 2022 at a dose of 12 mg once daily on days 1–14 of a 21-day cycle and was combined with camrelizumab from August to November 2022. The best documented response during this phase was transient radiographic shrinkage, with reduction of mediastinal lymph nodes and the left main bronchial lesion (**Figures 4A–D**). However, subsequent imaging in early 2023 showed gradual progression in the right lung(**Figures 4E, F**). Anlotinib was discontinued in May 2023 due to marked progression with right hilar mass and worsening atelectasis. After that, the patient underwent five cycles of gemcitabine from June to August 2023. During this phase, transient radiologic improvement was observed (**Figures 4G–L**). Nevertheless, a new left pleural effusion developed(**Figures 4I, J**). A subsequent chest CT performed on September 14, 2023, showed partial absorption of the pleural effusion(**Figures 4K, L**). Overall, the sequential multimodal treatments were generally tolerated, and no obvious severe treatment-related adverse reactions were documented during thoracic radiotherapy or during systemic treatment with camrelizumab-, anlotinib-, and gemcitabine-containing regimens.

In September 2023, the patient developed acute episodic chest tightness, worsening dyspnea, cyanosis, and tachycardia (up to 147 beats/min). He died one week later from multiple organ failure. The post-recurrence survival, measured from confirmed postoperative recurrence in July 2021 to death, was 26 months.

## 3 Discussion

NUT carcinoma is an ultra-rare, genetically defined malignancy driven by NUTM1 rearrangements, most commonly involving BRD4 or BRD3, which promote aberrant chromatin megadomain formation and transcriptional dysregulation, ultimately enforcing a poorly differentiated, highly aggressive phenotype (2, 7, 8). Thoracic NUT carcinoma is consistently associated with particularly unfavorable outcomes. Large retrospective cohorts have reported a median overall survival (OS) generally below 1 year, and thoracic primary site has been identified as an independent adverse prognostic factor (3, 5). In this context, the present case is particularly noteworthy because it involved primary pulmonary NUT carcinoma in an adolescent, followed by postoperative locoregional recurrence without distant metastasis, and was managed with sequential multimodal treatment across multiple lines, ultimately achieving 26 months of post-recurrence survival. Compared with the generally poor outcomes reported for thoracic NUT carcinoma, this clinical course suggests that prolonged survival may be achievable in select patients through individualized and carefully sequenced multimodal management.

Some factors may explain the relatively prolonged survival observed. First, the patient initially presented with resectable disease and underwent complete surgical resection (stage IB). Prior evidence indicates that gross total resection is among the strongest predictors of improved survival in NUT carcinoma, particularly when integrated into multimodal therapy (3, 5, 9). Second, at the time of first recurrence, the disease remained confined to the thorax without distant metastasis, allowing repeated locoregional interventions. The combination of radiofrequency ablation, definitive-dose thoracic radiotherapy (54Gy), and repeated bronchoscopic electrocautery debulking likely contributed to durable airway patency and local disease control. Such airway-directed strategies are rarely highlighted in large series but may be crucial in thoracic NUT carcinoma where endobronchial progression can rapidly lead to life-threatening obstruction (10, 11).

Immune checkpoint inhibitors (ICIs), particularly PD-1/PD-L1 inhibitors, have recently been explored in NUT carcinoma (12–16). Although evidence remains limited, case reports and small series have suggested that pembrolizumab or nivolumab may provide clinical benefit in metastatic or refractory disease, especially when combined with chemotherapy or radiotherapy (17–21). However, responses appear heterogeneous, potentially related to low tumor mutational burden, variable PD-L1 expression, and intrinsic biological features associated with BRD-NUT-driven tumor aggressiveness and therapeutic resistance (3, 4, 9, 21). Therefore, combination strategies have attracted increasing interest. In particular, anti-angiogenic agents such as anlotinib may enhance the activity of immunotherapy, while chemotherapy or radiotherapy may further augment antitumor effects through synergistic mechanisms (22–25).

In the present case, the patient experienced locoregional recurrence approximately two years after curative surgery. Subsequent treatment with camrelizumab combined with nab-paclitaxel resulted in disease stabilization for several months. Later, the combination of anlotinib and camrelizumab achieved radiographic reduction of mediastinal lymph nodes and the endobronchial lesion, suggesting a potential benefit of combined anti-angiogenic and immunotherapeutic approaches in this disease. However, the response was temporary, and progressive disease eventually occurred, highlighting the aggressive clinical course and therapeutic resistance characteristic of NUT carcinoma.

This case also raises translational considerations. MTAP loss, identified in the primary tumor, is increasingly recognized as a therapeutically relevant vulnerability in multiple solid tumors via induced dependence on PRMT5 and MAT2A pathways (26–28). While MTAP status has not been systematically evaluated in pulmonary NUT carcinoma cohorts, the detection of MTAP loss in this case suggests that future molecular profiling should include MTAP/CDKN2A assessment, and clinical trial enrollment should be considered whenever feasible (29, 30). In addition, this case illustrates the limitations of current standard therapies: cytotoxic chemotherapy (including taxanes and gemcitabine) provided only transient benefit, consistent with the generally short-lived responses reported in NUT carcinoma (3, 5). Meanwhile, targeted BET inhibition—one of the most rational disease-specific approaches—has demonstrated activity in early-phase trials but limited durability, and access remains restricted in many regions (4, 31).

Several limitations should be acknowledged. First, this is a single case and cannot establish causality regarding the contribution of any individual treatment component. Second, comprehensive molecular profiling (e.g., identification of the exact NUTM1 fusion partner, tumor mutational burden, and broader genomic alterations) was not performed due to financial limitations, limiting mechanistic interpretation. Third, longitudinal biomarkers such as circulating tumor DNA were not assessed, which could have provided insights into clonal evolution and resistance. Future directions should focus on establishing standardized diagnostic and treatment pathways for thoracic NUT carcinoma, promoting early referral to specialized centers, and expanding access to clinical trials. Rational combination strategies-such as BET inhibitors with HDAC inhibitors, ICIs, or anti-angiogenic agents-may improve response durability and should be evaluated prospectively. Additionally, MTAP-loss–directed therapies (PRMT5 or MAT2A inhibitors) represent a promising avenue for biomarker-driven treatment in this rare disease (26, 27).

Overall, this case supports the value of individualized multidisciplinary management and suggests a practical clinical implication: in selected patients with localized or intrathoracic recurrent primary pulmonary NUT carcinoma, particularly when recurrence remains confined to the thorax without distant metastasis, active locoregional control combined with sequential systemic treatment may help prolong survival.

## 4 Conclusion

Pulmonary NUT carcinoma remains a highly aggressive malignancy with historically poor survival outcomes, particularly in thoracic primary tumors. This case highlights the unique clinical value of a primary pulmonary NUT carcinoma in an adolescent that developed postoperative locoregional recurrence without distant metastasis and achieved 26 months of post-recurrence survival through individualized, multidisciplinary, and sequential multimodal management. Early complete surgical resection, aggressive locoregional control at recurrence, and the strategic integration of systemic therapies, particularly immunotherapy-based combinations with chemotherapy and anti-angiogenic agents, may collectively contribute to extended disease control, even in the absence of access to BET inhibitors.

The presence of MTAP loss in this case further highlights the importance of comprehensive molecular profiling and suggests potential avenues for future biomarker-driven therapies. Given the rarity of pulmonary NUT carcinoma, collaborative research efforts and prospective clinical trials are urgently needed to clarify the role of immunotherapy within multimodal treatment strategies and to improve outcomes in this devastating disease.

## Data Availability

The original contributions presented in the study are included in the article/supplementary material. Further inquiries can be directed to the corresponding authors.

## References

[1] French CA . The importance of diagnosing NUT midline carcinoma. Head Neck Pathol. (2013) 7:11–6. doi: 10.1007/s12105-013-0428-1. PMID: 23463074 PMC3597165

[2] French CA . NUT carcinoma: Clinicopathologic features, pathogenesis, and treatment. Pathol Int. (2018) 68:583–95. doi: 10.1111/pin.12727. PMID: 30362654

[3] Bauer DE Mitchell CM Strait KM Lathan CS Stelow EB Luer SC . Clinicopathologic features and long-term outcomes of NUT midline carcinoma. Clin Cancer Res. (2012) 18:5773–9. doi: 10.1158/1078-0432.ccr-12-1153. PMID: 22896655 PMC3473162

[4] Stathis A Zucca E Bekradda M Gomez-Roca C Delord JP de La Motte Rouge T . Clinical response of carcinomas harboring the BRD4-NUT oncoprotein to the targeted bromodomain inhibitor OTX015/MK-8628. Cancer Discov. (2016) 6:492–500. doi: 10.1158/2159-8290.cd-15-1335. PMID: 26976114 PMC4854801

[5] Chau NG Ma C Danga K Al-Sayegh H Nardi V Barrette R . An anatomical site and genetic-based prognostic model for patients with nuclear protein in testis (NUT) midline carcinoma: Analysis of 124 patients. JNCI Cancer Spectr. (2020) 4:pkz094. doi: 10.1093/jncics/pkz094. PMID: 32328562 PMC7165803

[6] Ge G Jiang X Tian X Zhou Y Cao G . The role of Salvia miltiorrhiza compounds in hepatocellular carcinoma: a preliminary investigation based on computational analysis and liquid chromatography-tandem mass spectrometry. Curr Pharm Anal. (2025) 21:249–64. doi: 10.1016/j.cpan.2025.04.001. PMID: 38826717

[7] Alekseyenko AA Walsh EM Wang X Grayson AR Hsi PT Kharchenko PV . The oncogenic BRD4-NUT chromatin regulator drives aberrant transcription within large topological domains. Genes Dev. (2015) 29:1507–23. doi: 10.1101/gad.267583.115. PMID: 26220994 PMC4526735

[8] Schwartz BE Hofer MD Lemieux ME Bauer DE Cameron MJ West NH . Differentiation of NUT midline carcinoma by epigenomic reprogramming. Cancer Res. (2011) 71:2686–96. doi: 10.1158/0008-5472.can-10-3513. PMID: 21447744 PMC3070805

[9] Chau NG Hurwitz S Mitchell CM Aserlind A Grunfeld N Kaplan L . Intensive treatment and survival outcomes in NUT midline carcinoma of the head and neck. (2016) 122:3632–40. doi: 10.1002/cncr.30242 PMC5361614 27509377

[10] Qu J Chen Z Zhu Y Huang J Shen Q . Pulmonary nuclear protein in testis (NUT) carcinoma: clinical, molecular characteristics, and treatment strategies. BMC Cancer. (2025) 25:196. doi: 10.1186/s12885-025-13593-3. PMID: 39905380 PMC11792297

[11] Kloker LD Sidiras M Flaadt T Brecht IB Deinzer CKW Groß T . Clinical management of NUT carcinoma (NC) in Germany: Analysis of survival, therapy response, tumor markers and tumor genome sequencing in 35 adult patients. Lung Cancer (Amsterdam Netherlands). (2024) 189:107496. doi: 10.1016/j.lungcan.2024.107496. PMID: 38301600

[12] Chandpa HH Gupta A Naskar S Meena J . Nanoparticles engineering strategies for lymph-node targeted cancer immunotherapy. Nano Trends. (2025) 11:100128. doi: 10.1016/j.nwnano.2025.100128. PMID: 38826717

[13] Lin A Zheng K Jiang A Huang X Wang Q Hajdu A . The evolving landscape of immunotoxicity: Charting mechanisms and future strategies for immune checkpoint inhibitor adverse events. Med Res. (2025) 1:322–58. doi: 10.1002/mdr2.70019. PMID: 41531421

[14] Wang J Chen Z Liu W Xu Z Liu H Li Y . Harnessing plant-derived natural compounds to target ferroptosis, pyroptosis, immune modulation and renin-angiotensin system in renal cell carcinoma. (2025) 26:. doi: 10.1177/14703203251386309

[15] Liang L Li Y Jiao Y Zhang C Shao M Jiang H . Maprotiline prompts an antitumour effect by inhibiting PD-L1 expression in mice with melanoma. Curr Mol Pharmacol. (2024) 17:e18761429259562. doi: 10.2174/0118761429259562230925055749. PMID: 37982288

[16] Kohli AS Sanyal S Kaushal RS Dwivedi M . An insight into immunological therapeutic approach against cancer: Potential anti-cancer vaccines. Curr Genomics. (2025) 26:175–90. doi: 10.2174/0113892029319505240821063238. PMID: 40433416 PMC12105320

[17] Lin A Che C Chen L Shi W Wong HZH Luo P . Immune checkpoint modulators in cancer immunotherapy from inhibition to activation. Curr Proteomics. (2025) 22:100056. doi: 10.1016/j.curpro.2025.100056. PMID: 38826717

[18] Zhang J Mao Y Zhang Y Wang X Yin W Ding L . Efficacy and safety of PD-1/PD-L1 inhibitors for relapsed/metastatic nasopharyngeal carcinoma: A systematic review and meta-analysis. Lett Drug Design Discov. (2025) 22:100128. doi: 10.1016/j.lddd.2025.100128. PMID: 38826717

[19] Sanchez Becerra MV Escudero Iriarte C Travert C Tian TV Besse B . Promising response to lurbinectedin in NUT carcinoma: a case report and review of emerging therapeutic strategies. Ann Oncol. (2025) 36:225–7. doi: 10.1016/j.annonc.2024.10.008. PMID: 39396757

[20] Liu Y Yuan C Sun J Zhang J Lin L Gu J . NUT carcinoma of the head and neck: Clinicopathologic and molecular analysis of 18 cases. Head Neck Pathol. (2025) 19:122. doi: 10.1007/s12105-025-01853-4. PMID: 41160255 PMC12572440

[21] Nakamura T Yoshida T Yoshida A Tateishi A Shinno Y Matsumoto Y . Immune checkpoint inhibitors in nuclear protein in testis carcinoma treatment: evidence of limited clinical benefits from a case series. Jpn J Clin Oncol. (2026). doi: 10.1093/jjco/hyag025. PMID: 41671149

[22] Li M Wang C Liu H Xu Z Liu Y . MiRNAs in cancers and renin-angiotensin-aldosterone system: Implications for chemoradiotherapy resistance. (2025) 26:. doi: 10.1177/14703203251367136

[23] Mou F Sun Q Ye Z . Advances in the combination of immunotherapy and radiotherapy for microsatellite-stable metastatic colorectal cancer. Curr Proteomics. (2025) 22:100049. doi: 10.1016/j.curpro.2025.100049. PMID: 38826717

[24] Shen G Zheng F Ren D Du F Dong Q Wang Z . Anlotinib: a novel multi-targeting tyrosine kinase inhibitor in clinical development. J Hematol Oncol. (2018) 11:120. doi: 10.1186/s13045-018-0664-7. PMID: 30231931 PMC6146601

[25] Xu Z Zhou H Li T Yi Q Thakur A Zhang K . Application of biomimetic nanovaccines in cancer immunotherapy: A useful strategy to help combat immunotherapy resistance. Drug Resist Update. (2024) 75:101098. doi: 10.1016/j.drup.2024.101098. PMID: 38833804

[26] Kryukov GV Wilson FH Ruth JR Paulk J Tsherniak A Marlow SE . MTAP deletion confers enhanced dependency on the PRMT5 arginine methyltransferase in cancer cells. Science. (2016) 351:1214–8. doi: 10.1126/science.aad5214. PMID: 26912360 PMC4997612

[27] Mavrakis KJ McDonald ER 3rd Schlabach MR Billy E Hoffman GR . Disordered methionine metabolism in MTAP/CDKN2A-deleted cancers leads to dependence on PRMT5. Science. (2016) 351:1208–13. doi: 10.1126/science.aad5944. PMID: 26912361

[28] Rodon J Prenen H Sacher A Villalona-Calero M Penel N El Helali A . First-in-human study of AMG 193, an MTA-cooperative PRMT5 inhibitor, in patients with MTAP-deleted solid tumors: results from phase I dose exploration. Ann Oncol. (2024) 35:1138–47. doi: 10.1016/j.annonc.2024.08.2339. PMID: 39293516

[29] Pulous FE Steurer B Pun FW Zhang M Ren F Zhavoronkov A . MAT2A inhibition combats metabolic and transcriptional reprogramming in cancer. Drug Discov Today. (2024) 29:104189. doi: 10.1016/j.drudis.2024.104189. PMID: 39306235

[30] Belmontes B Slemmons KK Su C Liu S Policheni AN Moriguchi J . AMG 193, a clinical stage MTA-cooperative PRMT5 inhibitor, drives antitumor activity preclinically and in patients with MTAP-deleted cancers. Cancer Discov. (2025) 15:139–61. doi: 10.1158/2159-8290.cd-24-0887. PMID: 39282709 PMC11726016

[31] Piha-Paul SA Hann CL French CA Cousin S Brana I Cassier PA . Phase 1 study of Molibresib (GSK525762), a bromodomain and extra-terminal domain protein inhibitor, in NUT carcinoma and other solid tumors. JNCI Cancer Spectr. (2020) 4:pkz093. doi: 10.1093/jncics/pkz093. PMID: 32328561 PMC7165800

